# Supplementary material for: Experiential Culinary, Nutrition and Food Systems Education Improves Knowledge and Confidence in Future Health Professionals
Source: Nutrients. 2023 Sep 15;15(18):3994. doi: 10.3390/nu15183994 (PMC10535872; doi:10.3390/nu15183994)
Supplement: Supplementary file 1 [file nutrients-15-03994-s001.zip › nutrients-2603972-supplementary.pdf]

Food Matters for Health Professionals Pre- and Post-Course Survey Questions

Demographics

1. Are you a clinician or student?
    a. Clinician
    b. Student
    c. Both
2. Are you attending the CME course or the CSPH 5905 course?
    a. CME course
    b. 6 week CSPH 5905
3. What is your gender?
    a. Male
    b. Female
    c. Other
    d. Prefer not to answer
4. Program of study or clinical area [answer: free text]
5. If clinic, # years in practice [answer: number]

Personal Habits and Approach to Food

6. Please rate the following questions on a scale of 1 (poor) to 5 (excellent):
    a. The nutritional quality of your daily food choices
    b. Your ability to prepare nutritious meals/snacks
    c. Your overall feeling of satisfaction with your daily food choices from a taste perspective
    d. The availability of monetary resources to eat a nutritionally balanced diet
    e. Your overall wellbeing
    f. Your overall health

Knowledge/Ability Related to Food and Culinary Practices

7. Please rate your level of knowledge and ability [answers were selected from a five-point Likert scale from 1 (strongly disagree) to 5 (strongly agree), with intermediate choices of 2 (disagree), 3 (neutral), and 4 (agree)]
    a. I know how to use a knife properly.
    b. I know how to plan, shop for and prepare a nutritionally balanced meal for myself/my family.
    c. I can define/explain the macro nutrients and know where to find them in food.
    d. I know how to read nutrition labels on the foods I eat to be able to understand the significance of calories, sodium, sugar and fat.
    e. I know how to budget my time to prepare and eat nutritionally balanced meals for myself.
    f. I know how to stock my kitchen/pantry with staple items to support the preparation of tasty and nutritious food.
    g. I know how to connect with peers and community to support my healthy eating.
    h. I am comfortable using at least 3 of the following cooking techniques with raw ingredients (blanch, steam, roast, saute, bake, braise).
    i. I know how to safely prepare a variety of raw, unprocessed foods as to not cause food borne illnesses from raw meats, fresh produce.
    j. I know how to safely store prepared foods.

8. I have sufficient knowledge to discuss each of the following diet styles with a patient or client [answers were selected from a five-point Likert scale from 1 (poor) to 5 (excellent), with intermediate choices of 2, 3, and 4]
    a. Vegetarian
    b. Vegan
    c. Paleo
    d. Mediterranean
    e. Gluten-free
    f. Low sodium
    g. Reduced sugar
    h. Lactose (dairy) intolerance
9. I have sufficient knowledge and skills to help a patient adapt a recipe to each specific diet [answers were selected from a five-point Likert scale from 1 (poor) to 5 (excellent), with intermediate choices of 2, 3, and 4]
    a. Vegetarian
    b. Vegan
    c. Paleo
    d. Mediterranean
    e. Gluten-free
    f. Low sodium
    g. Reduced sugar
    h. Lactose (dairy) intolerance

Knowledge in Relation to Clinical Practice

10. Please rate your level of knowledge in the following areas [answers were selected from a five-point Likert scale from 1 (poor) to 5 (excellent), with intermediate choices of 2, 3, and 4]
    a. I can identify at least 5 chronic diseases that are impacted by diet.
    b. I have the ability to link certain dietary patterns to pre-diabetes, diabetes, metabolic syndrome, obesity and cardiovascular disease risk-factors.
    c. I have an understanding of gut health and how it is influenced by food/dietary patterns.
    d. I understand the health benefits and effects of a whole foods, plant-rich diet.
    e. I understand the difference between types of fats and the role of each in a health-promoting dietary pattern.

Clinical Application

11. Please rate your practices around culinary nutrition in the clinical setting [answers were selected from a five-point Likert scale from 1 (strongly disagree) to 5 (strongly agree), with intermediate choices of 2 (disagree), 3 (neutral), and 4 (agree)]
    a. I am able to recommend dietary modifications with a patient/client with chronic, diet-related disease.
    b. I am able to guide a patient/client through steps of dietary changes and food preparation techniques.
    c. I appreciate the role of various members of the healthcare team in providing patient education and interventions around food, nutrition and cooking.
    d. I believe it is important to address nutrition with patients/clients on a regular basis, and make even a small amount of time to do so.
